# Targeting of Non-Classical Human Leukocyte Antigens as Novel Therapeutic Strategies in Cancer

**DOI:** 10.3390/cancers16244266

**Published:** 2024-12-22

**Authors:** Javier David Benitez Fuentes, Jorge Bartolome Arcilla, Kauzar Mohamed Mohamed, Alfonso Lopez de Sa, Alicia de Luna Aguilar, Kissy Guevara-Hoyer, Pablo Ballestin Martinez, Antonio David Lazaro Sanchez, Edgardo D. Carosella, Alberto Ocaña, Silvia Sánchez-Ramon

**Affiliations:** 1 Department of Medical Oncology, Elche General University Hospital, 03203 Elche, Spain; 2 Department of Medical Oncology, Hospital Clinico San Carlos, Instituto de Investigación Sanitaria San Carlos (IdISSC), 28040 Madrid, Spain; jorge.bartolome@salud.madrid.org (J.B.A.); alfonso.lopezdesa@salud.madrid.org (A.L.d.S.); pablo.ballestin@salud.madrid.org (P.B.M.); 3 Experimental Therapeutics in Cancer Unit, Instituto de Investigación Sanitaria San Carlos (IdISSC), 28040 Madrid, Spain; 4 Department of Immunology, IML and IdISSC, Hospital Clinico San Carlos, 28040 Madrid, Spain; kauzar.mohamed@salud.madrid.org (K.M.M.); kissy.guevara@salud.madrid.org (K.G.-H.); ssramon@salud.madrid.org (S.S.-R.); 5 Department of Medical Oncology, Hospital General Universitario Morales Meseguer, 30008 Murcia, Spain; aliciamilagro.deluna@carm.es; 6 Department of Immunology, Ophthalmology and ENT, School of Medicine, Complutense University, 28040 Madrid, Spain; 7 Department of Medical Oncology, Hospital 12 de Octubre, 28041 Madrid, Spain; 8 Department of Medical Oncology, Santa Lucia General University Hospital, 30202 Cartagena, Spain; antoniod.lazaro@carm.es; 9 CEA, DRF-Institut de Biologie François Jacob, Service de Recherches en Hémato-Immunologie, Hôpital Saint-Louis, 75010 Paris, France; edgardo.carosella@cea.fr; 10 U976 HIPI Unit, IRSL, Université Paris, 75006 Paris, France; 11 START Madrid-Fundación Jiménez Díaz (FJD) Early Phase Program, Fundación Jiménez Díaz Hospital, 28040 Madrid, Spain

**Keywords:** human leukocyte antigen, non-classical HLAs, solid cancers, HLA-E, HLA-F, HLA-G, HLA-H, immunotherapy

## Abstract

Human leukocyte antigens (HLAs) are essential regulators of immune responses against cancer, with classical HLAs well-documented for their role in tumor recognition and immune surveillance. In recent years, non-classical HLAs—including HLA-E, HLA-F, HLA-G, and HLA-H—have emerged as critical players in the immune landscape of cancer due to their diverse and less conventional functions in immune modulation. These molecules exhibit unique mechanisms that enable tumors to escape immune detection, promote tumor progression, and contribute to therapeutic resistance. This review provides a comprehensive examination of the current understanding of non-classical HLAs in solid cancers, focusing on their specific roles in shaping the tumor microenvironment and influencing immune responses. By analyzing how HLA-E, HLA-F, HLA-G, and HLA-H modulate interactions with immune cells, such as T cells, natural killer cells, and antigen-presenting cells, we highlight key pathways through which these molecules contribute to immune evasion and metastasis. Additionally, we review promising therapeutic strategies aimed at targeting non-classical HLAs, including emerging immunotherapies that could potentially enhance cancer treatment outcomes by reversing immune suppression within tumors. Understanding the influence of these non-classical HLAs in solid cancers may offer new insights into cancer immunology and may lead to the development of innovative and more effective immunotherapeutic approaches. This review underscores the importance of non-classical HLAs as potential therapeutic targets, providing a necessary foundation for future studies in the evolving field of cancer immunotherapy.

## Simple Summary

Non-classical human leukocyte antigens (HLAs) are molecules that help control the immune system’s response to cancer. While classical HLAs have been studied for their role in detecting and fighting tumors, non-classical HLAs—specifically HLA-E, HLA-F, HLA-G, and HLA-H—are less understood but appear to play unique roles in helping tumors evade the immune system. This review examines how these non-classical HLAs contribute to cancer progression and discusses their potential as new targets for cancer treatments. By exploring the specific functions of HLA-E, HLA-F, HLA-G, and HLA-H in various cancers, the authors aim to highlight their importance in the immune system’s interaction with tumors.

## 1. Introduction

Human Leukocyte Antigens (HLAs) are essential for immune recognition, helping to discern self from non-self organisms. Discovered over six decades ago, they are a central part of the process of antigen presentation to immune effector cells [1]. While classical HLAs, predominantly encoded by HLA-A, HLA-B, and HLA-C genes, have enjoyed extensive scrutiny for their involvement in tumor immunosurveillance, in anti-tumor immunity, and in immune escape mechanisms, the less understood area of non-classical HLAs has also garnered attention [2,3,4,5]. With this scrutiny, it is becoming increasingly apparent that non-classical HLAs, including HLA-E, HLA-G [6], HLA-F [7], and HLA-H [8], exert roles that transcend conventional antigen presentation.

Aberrant expression of non-classical HLAs within the tumor microenvironment (TME) can result from various factors, including complex immunoregulatory signals and intratumoral heterogeneity [9,10,11,12]. The distinct antigens non-classical HLAs present further influence the interaction between tumor cells and the immune system, potentially altering immune recognition and response mechanisms [11]. Additionally, soluble forms of these non-classical HLAs hold potential as prognostic biomarkers [9]. Their expression profiles in specific cancer types, such as gastric cancer, pancreatic cancer, colorectal cancer [9], breast cancer, melanoma, and renal cell carcinoma, suggest potential roles in modulating the tumor–immune interface [10]. Studies point to their involvement in regulating the activity of immune effectors, including Natural-Killer (NK) cells and T cells, which can impact anti-tumor immune responses [11]. The interplay between non-classical HLAs and solid cancers could provide new insights into the balance between immune surveillance and immune tolerance within the TME [12].

Taking advantage of their potential to influence immune responses and tumor behavior, researchers are investigating therapeutic strategies targeting these proteins, for example, through the use of monoclonal antibodies (mAB) that can act as immune checkpoint inhibitors (ICIs) to modulate immune evasion mechanisms orchestrated by some of them [13,14,15,16]. For instance, in cancers marked by the overexpression of non-classical HLAs, like HLA-G or HLA-E, interventions aimed at blocking their interactions with immune receptors could potentially restore immune surveillance and amplify anti-tumor responses. This holds promise in enhancing the efficacy of immunotherapies by dismantling immune escape mechanisms fostered by these HLAs [11,12,13,14,15,16,17]. In this context, several emerging drugs, which will be reviewed in this manuscript, are under development against this axis [16,17,18,19].

We aim to review the different roles of non-classical HLAs in solid cancer immunology and point out their potential as new targets for immunotherapy treatments.

## 2. Human Leukocyte Antigens and Immune Surveillance

The HLA is a gene family clustered in chromosome 6. This family is constituted of more than 200 genes that codify three HLA subgroups: class I, class II, and class III [20]. HLA-I and HLA-II are genetically located within the same locus and share a similar structure and function, that is mainly the presentation of peptides at the cell’s surface to be recognized by the immune system. Proteins codified by these genes are key players in the immune response to pathogens and in the development of self-tolerance [21,22]. These proteins present antigens in the form of peptides to the immune system, so it can recognize its own antigens and non-self-ones, thus enabling a humoral and cellular immune response [23]. In order for T cells to be activated, they need to recognize specific antigens in the surface-expressed HLA-I and II molecules. In addition, these molecules act as ligands for NK cell receptors, influencing the activation or inhibition of these cells [24]. HLA-III refers to a group of genes within the major histocompatibility complex (MHC) class III region on chromosome 6 which are involved in various other immune functions [20]. They encode components such as complement proteins, cytokines, and heat shock proteins, crucial for immune regulation, inflammation, and stress response [20].

### 2.1. HLA-I

HLA-I molecules present peptides derived from intracellular proteins, including self-proteins, as well as those originating from pathogens such as viruses or intracellular bacteria, to CD8+ T cells. In doing so, they allow the immune system to survey the intracellular environment and detect abnormal changes, including infections or malignant transformations [25,26]. This endogenous antigen presentation pathway ensures that any alterations within the cell are promptly displayed on its surface, enabling immune effectors to respond swiftly [22,25].

To accomplish this, HLA-I follows a series of coordinated steps. First, intracellular proteins intended for degradation are tagged with ubiquitin, marking them for breakdown by the proteasome into small peptide fragments. Second, these peptides are transported into the endoplasmic reticulum (ER) by the transporter associated with antigen processing, which selectively shuttles peptides with suitable lengths and sequences [25]. Third, within the ER, a network of chaperone proteins (including calreticulin and tapasin) and the peptide-loading complex assists in loading these peptides onto HLA-I heavy chains bound to β2-microglobulin, ensuring the formation of a stable HLA-I/peptide complex [22,25]. Fourth, after achieving a proper fit, the stable HLA-I/peptide complex leaves the ER, passes through the Golgi apparatus, and is ultimately expressed on the cell surface [25,26]. If a peptide is suboptimal and fails to achieve stable binding, it is released and eventually returned to the cytosol for further degradation, thus maintaining a high standard of peptide quality control [27,28].

While HLA-II molecules are typically expressed by specialized antigen-presenting cells (APCs) to present exogenous antigens, HLA-I is widely expressed in nearly all nucleated cells. Its primary role is to present endogenously derived peptides, allowing CD8+ cytotoxic T cells to continually monitor cellular health and identify infected or malignant cells [25]. In addition to guiding CD8+ T cells, HLA-I molecules also interact with NK cells, providing inhibitory signals when displaying self-peptides and thereby contributing to the maintenance of self-tolerance and immune homeostasis [26].

### 2.2. HLA-II

HLA-II molecules specialize in presenting exogenous antigens to CD4+ T cells, allowing the immune system to detect and respond to pathogens that are outside the cell. Primarily expressed by APCs, such as DCs, macrophages, and B cells [29], these molecules capture peptides derived from external pathogens and display them on the cell surface, where they can be recognized by CD4+ T cells [29,30,31]. In this process, extracellular proteins are first internalized by APCs through endocytosis or phagocytosis. These internalized proteins are then degraded into peptide fragments within acidified endosomal and lysosomal compartments, ensuring only properly processed antigens are presented [22,23,29,31,32]. Meanwhile, newly synthesized HLA-II molecules assemble in the ER bound to the invariant chain (Ii), which occupies the peptide-binding groove, preventing premature peptide loading. This complex is transported from the ER to specialized endosomal compartments, where the invariant chain is cleaved, leaving a small fragment known as CLIP (class II-associated invariant chain peptide) occupying the binding groove. HLA-DM, a non-classical MHC class II molecule, then removes CLIP and facilitates the loading of the appropriate peptide fragments onto HLA-II. Once a stable HLA-II/peptide complex is formed, it is trafficked to the APC surface [22,23,29,31,32].

When CD4+ T cells recognize the HLA-II/peptide complexes displayed on the APC surface, they are activated and orchestrate a variety of immune responses. Activated CD4+ T cells provide essential help for B cells to produce antibodies, support macrophage activation, and enhance CD8+ T cell responses, thereby shaping both humoral and cell-mediated immunity [29,31,33]. This exogenous antigen presentation pathway ensures that the immune system can detect and respond to extracellular pathogens.

Additionally, HLA-II molecules play a significant role in maintaining immune tolerance and preventing autoimmunity by presenting self-peptides to T cells. Under inflammatory conditions, cells not typically considered APCs, such as fibroblasts or endothelial cells, can be induced to express HLA-II, expanding the range of cells capable of presenting exogenous antigens [29,34].

**Figure 1:**
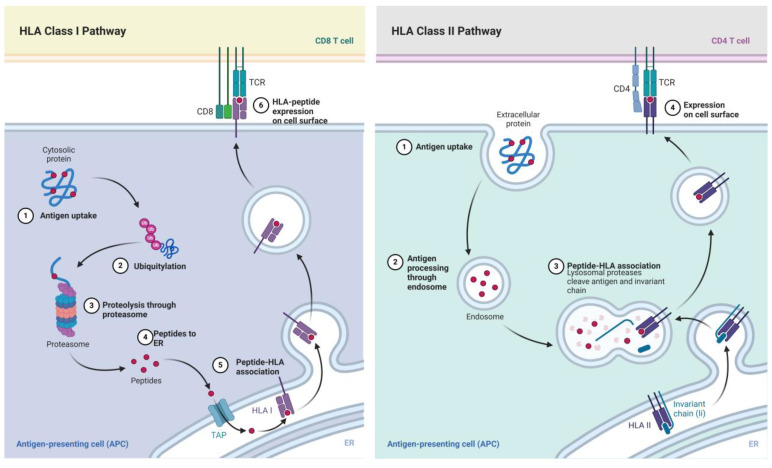
Schematic overview of HLA-I and HLA-II antigen processing and presentation pathways.

A schematic representation of both HLA-I and HLA-II antigen processing and presentation pathways is provided in Figure 1, offering a visual summary of the key steps involved in displaying intracellular and extracellular peptides to CD8+ and CD4+ T cells, respectively.

### 2.3. HLA-III

The HLA-III region is an important genomic segment located within the MHC on chromosome 6 [20,35]. It is distinct from the HLA class I and class II regions in both structure and function. Unlike the HLA class I and class II genes, which primarily encode cell surface proteins involved in presenting peptides, the class III region contains a variety of genes with broader roles, including inflammatory response mediators and components of the complement system. Some key genes in this region include those encoding components of the complement pathway and cytokines, such as tumor necrosis factors, which are crucial for immune and inflammatory responses [20,35]. HLA-III is also characterized by lower genetic variability compared to the highly polymorphic class I and II genes, but it still plays a significant role in immune function and susceptibility to diseases. Studies indicate that variations in this region can influence the risk for different diseases [35,36].

## 3. Non-Classical Human Leukocyte Antigens: Types and Functions

The existence of non-classical HLA-I (HLA-Ib: HLA-E, F, G, and H) has gained great interest due to its particular role in the modulation of the immune response to cancer. Structurally, non-classical HLA-I proteins show less polymorphism than classical HLA-I molecules. They are known to have the capacity to present different classes of intracellular antigens that are afterward recognized by innate and adaptive immune receptors [20,37]. However, it must be noted that non-classical HLA-I may have mostly inhibitory effects on immune response through the activation of inhibitory receptors [20]. Furthermore, certain HLA-I molecules have non-immunological functions [1].

### 3.1. HLA-E

HLA-E is an oligomorphic protein with a wide expression across virtually every tissue; however, it is predominantly expressed by immune cells and endothelial cells. It has been shown that HLA-E expression correlates with the expression of HLA-Ia proteins [11,20,38]. It can participate in both the activation and inhibition of immune responses, and its activity is of particular interest in the regulation of NK cells through the stimulatory receptor CD94/NKG2C and the inhibitory receptor CD94/NKG2A [20,39].

### 3.2. HLA-F

HLA-F is a mainly intracellular protein with low levels of polymorphism that is expressed exclusively on lymphocyte B cells, monocytes, and trophoblasts, while its expression can be induced in many immune cells by NFκB and IFNγ [40,41]. HLA-F is capable of binding to HLA-I heavy chains and migrating to the cell membrane, where these complexes function as inhibitory ligands for immune cell receptors [11,40,41,42]. Its main function is to regulate the immune system by binding to the inhibitory immunoglobulin-like transcript 2 (ILT-2) and ILT-4 receptors, which are expressed in many immune cells, as well as with KIR receptors, present in NK cells [11,40,41].

### 3.3. HLA-G

The role of HLA-G was first studied in cytotrophoblasts and has been found to play a role in inducing maternal tolerance to the fetus. It is scarcely expressed throughout the body but is present in certain immune-rich tissues such as the maternal-fetal interface of the placenta or pancreatic islet β cells [43,44], as well as in hematopoietic cells. Moreover, it shows a limited polymorphism and peptide diversity. Its activity is regulated by many stimuli like IL-10, IFN-γ, oxidative stress or radiation [11]. Its function is mainly immunosuppressive, being capable of limiting cellular processes like the cytotoxic functions of CD8+ T cells, the proliferative process of CD4+ T, DC, or NK cells, or the chemokine receptor expression of T cells, through its ability to bind to many inhibitory proteins [44]. Likewise, HLA-G is capable of inducing apoptosis of T Lymphocytes and NK cells [45].

Its primary transcript undergoes alternative splicing, resulting in the production of at least seven mRNA variants that encode four membrane-bound isoforms (HLA-G1 to HLA-G4) and three soluble isoforms (HLA-G5 to HLA-G7) [46]. These isoforms exhibit one, two, or three extracellular domains (α1, α2, and α3). Recently, an eighth isoform, HLA-G∆α1, has been identified, which uniquely lacks the α1 extracellular domain. HLA-G∆α1 has been detected in clear cell renal cell carcinomas and trophoblasts. Notably, HLA-G∆α1 does not associate with β2-microglobulin, potentially lacks the peptide-binding groove necessary for presenting peptides to T cells, and exhibits immunostimulatory properties towards peripheral blood NK and T cells. This contrasts with all other known HLA-G isoforms, which typically function as inhibitory immune checkpoint molecules [47].

HLA-G can be secreted either as soluble sHLA-G or packaged within extracellular vesicles (EVs) and can be detected in diverse body fluids including plasma, ascites, and pleural exudate [48]. Recent research indicates that tumor cells, cytotrophoblast cells, and mesenchymal stem cells are capable of releasing EVs carrying HLA-G. These EVs play a significant role in modulating the TME and exerting immunosuppressive functions [49].

### 3.4. HLA-H

HLA-H is primarily recognized for its role in regulating iron metabolism [50,51]. It modulates the interaction between transferrin and its receptor. This modulation is crucial for maintaining iron homeostasis in the body. Dysregulation in HFE can lead to conditions like hereditary hemochromatosis [50]. Beyond its role in iron regulation, HLA-H is also implicated in several functions related to the immune system [52]. Recent studies suggest that HLA-H could affect the immune system by modulating the expression and function of other non-classical MHC class I molecules, such as HLA-E [52]. This modulation could impact how NK cells and cytotoxic T cells respond to infected or malignant cells, thereby acting on immune surveillance and tolerance [52].

## 4. Association Between Non-Classical HLAs and Solid Cancers

Research into the role of non-classical MHC molecules in human oncogenesis is relatively recent but is a rapidly expanding field [38]. HLA-G is presumably the most studied non-classical HLA in solid tumors [53]. Data regarding HLA-E have also been reported in several types of solid cancer whereas information regarding HLA-F is still scarce [54] and almost absent for HLA-H.

### 4.1. HLA-G

High HLA-G expression in solid cancers has been associated with tumor size, advanced disease, tumor metastasis, and worse survival rates [9,55]. Overexpression of HLA-G was first found in melanoma samples with an absence in adjacent healthy tissues [56]. Several malignancies since then have associated HLA-G expression with outcome including lung cancer, melanoma, renal cancer, colon cancer, cervical cancer, endometrial carcinoma, oesophageal cancer, glioblastoma, gastric cancer, and breast cancer, among others [9,57]. Its expression in esophageal squamous cell carcinoma, for example, is associated with poorer prognosis, suggesting its potential as a diagnostic and prognostic marker [58]. Inter- and intra-tumoral heterogeneity of HLA-G expression has been observed in several studies of HLA-G at a transcription and protein level, either among patients with different types of neoplasm, or among patients with the same type of solid tumor [59,60].

These data align with findings indicating HLA-G’s role in the immune escape of cancer cells, correlating with clinical parameters in many tumors, thus underscoring its significance in cancer diagnosis and prognosis [9,61]. Although the primary function of HLA-G is to inhibit the immune response, its interaction with ILT4 may also create a favorable microenvironment for tumor growth. In non-small cell lung cancer (NSCLC) cell lines, stimulation with HLA-G fusion protein increases ILT4 expression and activates the extracellular signal-regulated kinase (ERK) signaling pathway. The co-expression of ILT4 and HLA-G is linked to increased lymph node invasion, advanced cancer stages, and reduced overall survival. This mutual regulation between HLA-G and ILT4 expression suggests the possibility of a feedback mechanism, although this has not been definitively established [62].

While extensive research has focused on the expression and clinical relevance of HLA-G in solid tumors, there remains a paucity of data regarding its expression and significance in liquid neoplasms. In hematological malignancies, tumor cells can express HLA-G receptors, such as ILT2. HLA-G appears to play an unexpected role by attenuating the proliferation of these neoplastic immune cells [63]. Specifically, HLA-G has demonstrated inhibitory effects on the proliferation of human B-cell lymphomas, myelomas, and B-cell leukemias expressing ILT2 receptors on their surfaces. This observation suggests a novel therapeutic avenue, utilizing the anti-proliferative properties of HLA-G to potentially curb tumor progression in hematological malignancies [53].

### 4.2. HLA-E

Elevated levels of HLA-E are present in various solid cancer types such as breast cancer, gynecologic cancers, liver cancer, non-small cell lung cancer (NSCLC), pancreas cancer, and colorectal cancer [64,65,66]. HLA-E surface expression varies depending on the availability of HLA class I molecules; an increased expression of HLA class I could involve a higher expression of HLA-E through the stabilization of HLA-E molecules [64]. The expression of HLA-E has also been detected in solid tumors with downregulated classical HLA class I surface expression. High levels of HLA-E with downregulated HLA class I molecules were associated with a significantly worse survival in serous ovarian carcinoma [65]. The expression of HLA-E in NSCLC has also been found to restrain the positive prognostic effect of stromal CD8+ tumor-infiltrating T cells, highlighting its complex role in immune modulation within the TME [66,67]. On the other hand, the expression of the HLA-E molecule on the surface could also be enhanced by the non-classical HLA molecule HLA-G, especially HLA-G1 and HLA-G3.

HLA-E polymorphism in solid cancer has been scarcely reported. While one study found a higher frequency of the HLA-E*01:03 allele in nasopharyngeal carcinoma patients compared to healthy controls [68], another study did not find significant differences [69].

### 4.3. HLA-F

The role of HLA-F in solid cancer is still unclear. High HLA-F surface expression has been associated with tumor size and a poor clinical outcome in breast cancer [70] and with cell invasion in gastric cancer patients [71]. Its prognostic significance appears to be variable. While it is associated with poor survival in NSCLC, suggesting its potential as a prognostic indicator [72], in other indications, like in gastric cancer, the association with prognosis is less clear, suggesting a more nuanced function across different cancer types [73]. Further studies are required to clarify its function.

## 5. Role of Non-Classical HLAs in Tumor Immune Escape

Multiple studies support the association between the progression of cancer and an altered expression of HLA class Ib molecules, particularly HLA-G, with a concomitant downregulation of classical HLAs [9,74,75].

### 5.1. HLA-G

**Figure 2:**
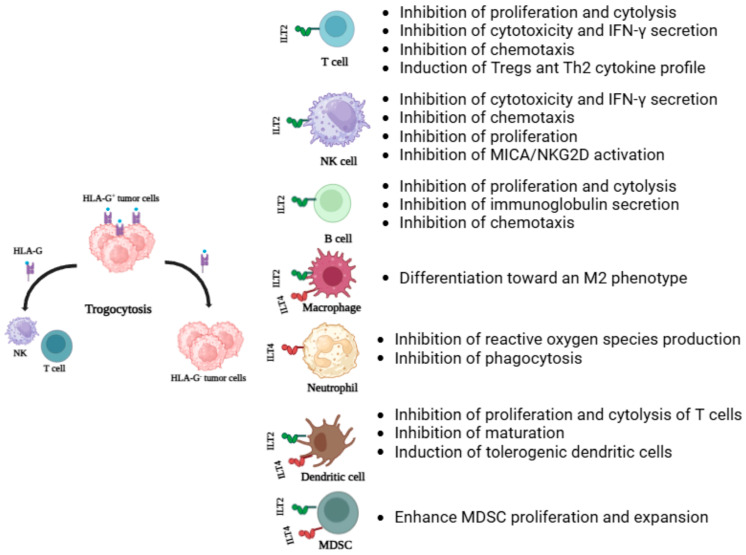
Role of HLA-G in tumor immune evasion. Tolerogenic functions of HLA-G through trogocytosis between HLA-G+ and HLA-G− tumoral cells and immune cells. HLA-G also performs suppressive function by binding to receptors expressed by different types of immune cells.

HLA-G is presumably the most distinct immunosuppressive molecule of the non-classical HLAs favoring tumor immune escape [9,75]. This molecule is able to execute this role through various strategies (Figure 2).

One mechanism is the interaction with inhibitory receptors that are present on T, B, NK, dendritic cells, and neutrophils, thus inhibiting their function that counteracts immune activation favoring tumor survival [76,77]. To date, numerous receptors for HLA-G ligand have been identified, such as immunoglobulin-like transcription receptor type 2 (ILT2/CD85j), ILT4/CD85d, killer cell immunoglobulin-like receptor 2DL (KIR2DL4/CD158d), CD160, and CD8. ILT2 is present on T, NK cells, and antigen presenting cells (APCs) while ILT4 is on APCs surface and neutrophils [76,77]. KIR2DL4 is exclusive of NK cells. The CD160 molecule is expressed by CD8+ and CD4+ subsets and CD56dim NK cells whereas CD8 is on CD8+ T cells and some NK cells. All these molecules present immunoreceptor tyrosine-based inhibition motifs (ITIMs) in their cytoplasmic domains which cause the inhibitory effects on target cells that include blocks of immune cell proliferation, cytotoxicity, differentiation, chemotaxis, and the induction of regulatory T cell (Tregs) [78]. Additionally, HLA-G induces the expansion of myeloid-derived suppressor cells (MDSCs), macrophage differentiation toward an M2 phenotype, and displace cytokine production toward a Th2 profile [79]. HLA-G could also enhance the expression of matrix metalloproteinases (MMPs) which are tumor metastasis-related factors favoring tumor progression [80].

An indirect immunosuppressive effect of HLA-G mediated by the induction of suppressor/regulatory cells has been described [81]. HLA-G-induced tolerogenic DCs can stimulate the generation of regulatory T cells [82]. Moreover, APCs bearing HLA-G induce T cell anergy and differentiation into suppressive cells [83].

HLA-G also performs suppressive functions throughout trogocytosis. Trogocytosis involves transferring cell-surface membrane proteins and membrane patches from one cell to another during contact [84]. This process has been described in T and B lymphocytes, NK cells, APCs, and tumor cells. HLA-G expressed by tumor cells can be acquired by immune cells which convert in HLA-G-positive cells reversing their function from effectors to regulatory cells. Consequently, this HLA-G-mediated immune evasion can also extend to nearby HLA-negative cells, thereby amplifying the tolerogenic effects [85].

### 5.2. HLA-E

The HLA-E molecule is attracting a gradually growing interest in tumor immunology. HLA-E binds peptides originated from the leader sequence of other HLA-class I molecules, namely HLA-A, HLA-B, HLA-C, and HLA-G [86]. Under normal conditions, HLA-E ligand interacts with the inhibitory receptor CD94/NKG2A carried by NK and a subset of CD8+ T cells. This interaction inhibits the cytotoxic activity of these cells against targets expressing HLA-class I molecules [86]. In tumor cells, HLA class I surface expression is downregulated [86]. As a consequence, a reduced number of HLA class I derived peptides are originated which result in low HLA-E expression, thus allowing lysis of tumoral cells by NK cells. Nevertheless, some malignant cells can evade immune surveillance by upregulation of HLA-E expression. This in turn leads to the inhibition of the cytolytic function of NK and T cells [87]. Other receptors for HLA-E are TCR of CD8+ T cells and CD160. As seen with HLA-G, this interaction turns into apoptosis of immune cells. This phenomenon was also observed with soluble HLA-E [88]. The binding of HLA-E to the TCR might also induce the generation of CD8+ Tregs [89].

**Figure 3:**
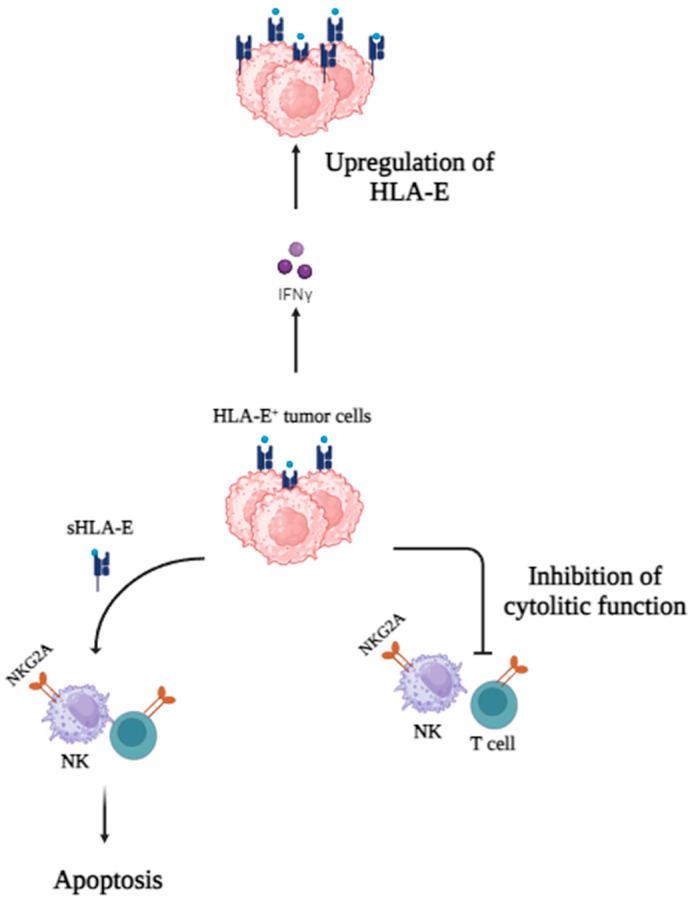
Role of HLA-E in tumor immune evasion. In the presence of IFN-γ, HLA-E is upregulated on tumor cells. The binding of HLA soluble (sHLA-E) to NKG2A receptor induces apoptosis of NK and CD8 T cells. The binding of HLA ligands on cancer cells’ surface to NKG2A inhibits the cytolytic functions of NK and CD8+ T cells.

Following INF-γ stimulation, an increased level of non-classical MHC class I molecules on the surface of tumoral cells was observed [90]. In the cancer niche, proinflammatory responses occur with high levels of INF-γ which may potentially result in negative feedback responses with the upregulation of non-classical HLA [90,91] (Figure 3).

### 5.3. HLA-F

**Figure 4:**
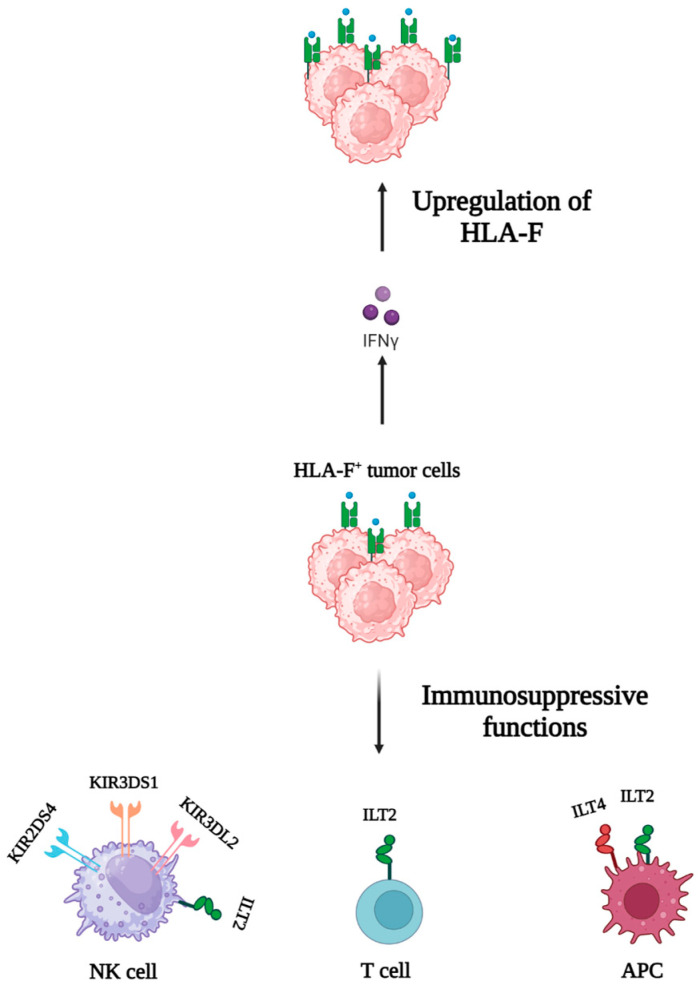
Role of HLA-F in tumor immune evasion. In the presence of IFN-γ, HLA-F is upregulated on tumor cells. The interaction of HLA-F with different receptors induces immunosuppressive functions.

HLA-F is considered the most elusive member of non-classical MHC molecules. Elevated HLA-F expression has been found in cancer cells [7]. The HLA-F ligand is able to bind to ILT-2, ILT-4, and various killer cell immunoglobulin-like receptors (KIR), including KIR3DS1 (three immunoglobulin domains and short cytoplasmic tail 1), KIR2DS4 (two immunoglobulin domains and short cytoplasmic tail 4), and KIR3DL2 (three immunoglobulin domains and long cytoplasmic tail 2), which triggers immunosuppressive functions on immune cells (Figure 4) [92]. HLA-F expression can be induced by IFN-γ, as seen with HLA-G and HLA-E [93].

## 6. Advances in Therapeutic Approaches Targeting Non-Classical HLAs

The current available standard of care immunotherapy targets adaptive immune system checkpoints enhancing T cell-mediated immunity. These adaptive immune system checkpoints are programmed death ligand-1 (PDL-1), programmed death-1 (PD-1), cytotoxic T-lymphocyte antigen 4 (CTLA4), and lymphocyte activation gene-3 (LAG-3) [94,95]. However, many patients will not benefit from this approach, or tumors will eventually progress to current therapies. Other alternative immune checkpoints are T cell immunoglobulin and mucin-domain containing (TIM)-3, T cell immune receptors with immunoglobulin and ITIM domains (TIGIT), and indoleamine 2,3-dioxygenase (IDO)-1, among several others [94,95].

Recent findings concerning non-classical HLAs and their role in tumor immune-evasion and progression, discussed above, places them as a potential target for the development of novel immunotherapeutics. Some molecules targeting these pathways are already being tested in phase III trials [95,96]. As we gather more information about the mechanisms concerning tumor immune escape, it seems that the combination of ICIs could lead to a deeper, more durable antitumor effect. In this scenario, HLA Ib-targeted therapies stand as potential partners that could enhance the antitumor activity of ICIs and other anticancer therapies. Table 1 summarizes clinical trials published or ongoing regarding non-classic HLA-related strategies [94,95].

### 6.1. HLA-E

HLA-E is the one non-classical HLA for which most progress has been made in terms of therapeutic actionability. As discussed above, HLA-E expression levels are increased in tumor cells as compared to healthy tissues [59,66,97], resulting in the inhibition of cytotoxic NK cells and a subset of CD8 T lymphocytes via its interaction with heterodimer NKG2A/CD94, expressed specifically in those cytotoxic immune populations [98]. NK cells not only are key as cytotoxic agents, but they also play a major role in antibody-mediated cellular cytotoxicity (ADCC), a known effector mechanism for many anticancer antibodies [99]. Moreover, NK cells recruit a subset of dendritic cells into solid tumors, stimulating crosstalk between the innate and adaptive immune systems [99].

Hence, targeting the NKG2A/HLA-E axis seems like a promising strategy for enhancing the antitumor immune response. While both ends of the NKG2A/HLA-E axis could be blocked, it has been preferred to target NKG2A as inhibition of HLA-E could prevent interaction with the activating NKG2C receptor [13]. Monalizumab (IPH2201, Innate Pharma/AstraZeneca) is a first-in-class humanized IgG4 antibody that binds to the NKG2A/CD94 receptor, blocking its interaction with HLA-E and enhancing NK and CD8 T cell activation [16]. In vitro studies showed that monalizumab alone promoted NK cell activity. Its combination with anti-PD-L1 had a synergistic effect, boosting NK cell and CD8 T cell effector functions. Moreover, the combination of the anti-EGFR mAB cetuximab with monalizumab increased NK cell ADCC activity in head and neck cancer cells in vitro [16].

Early clinical trials with monalizumab monotherapy were held with 58 gynecological cancer patients who were assessed in a phase I trial with monalizumab monotherapy [100]. The drug was well-tolerated, but the efficacy results were poor with stable disease as the best response in 39% of patients in part 1 and 18% of patients in part 2 [100]. Monalizumab monotherapy was also tested in a Phase II umbrella trial with refractory advanced head and neck squamous cell carcinoma (HNSCC) patients (UPSTREAM, NCT03088059) [101]. With an ORR of 0%, this cohort was closed in the interim futility analysis [101]. The drug is being tested with durvalumab in another cohort (I2) of the trial.

A phase I/II trial tested monalizumab in combination with anti-PD-L1 Durvalumab in patients with solid tumors [102]. While the effectiveness was moderate, the combination of monalizumab and durvalumab was well-tolerated, and promising signs of immune activation were noted in both the peripheral blood and the TME [102]. Combinations with anti-PD-L1 Durvalumab were also tested in patients with NSCLC. The COAST study (NCT03822351) tested the combination of monalizumab and durvalumab in unresectable stage III NSCLC after chemoradiation, showing encouraging results in terms of ORR and PFS versus Durvalumab alone [103]. This led to an ongoing Phase III study (PACIFIC-9, NCT05221840) in this population [96]. A combination of Monalizumab with durvalumab has also been tested in patients with NSCLC in the neoadjuvant setting in the NeoCOAST trial (NCT03794544) with promising results improving major pathologic response rates [104]. A Phase II multicenter trial is now being held in that setting (NeoCOAST-2, NCT05061550) [105]. Monalizumab plus Durvalumab is being tested in PD-1 therapy-resistant NSCLC patients in a Phase II study (NCT03833440) [106] and in the first line setting of small cell lung cancer (MOZART trial, NCT05903092) [107].

Another Phase Ib/II trial (NCT02643550) showed promising results with the combination of monalizumab and Cetuximab in advanced HNSCC patients [108], leading to a Phase 3 trial (INTERLINK-1, NCT04590963) [109]. However, the study was discontinued after an interim analysis as it did not meet the predefined threshold for efficacy. The MIMOSA trial (NCT04307329) delves further into the monalizumab-antibody synergy enhancing ADCC by combining it with trastuzumab [110]. In this Phase II trial, 11 metastatic HER2+ breast cancer patients were enrolled, showing no objective responses. The study was terminated after the primary endpoint of the trial was not met [110].

Preclinical data also suggest the ability of monalizumab to empower immune anti-cancer activity unchained by therapeutic cancer vaccines, setting a promising landscape for future investigations [111]. Also, intra-tumoral injection of monalizumab and allogenic NK cells is another approach being tested in the preclinical setting [112].

In summary, it seems that strategies aiming to enhance ADCC activity by combining with antibodies like trastuzumab or cetuximab, have not reached the desired effects. On the other hand, in clinical scenarios where primary naïve tumors are treated after or concomitantly with chemotherapy, the release of antigens could enhance the activity of NKG2A antibodies, showing promising signs of activity.

**Figure 5:**
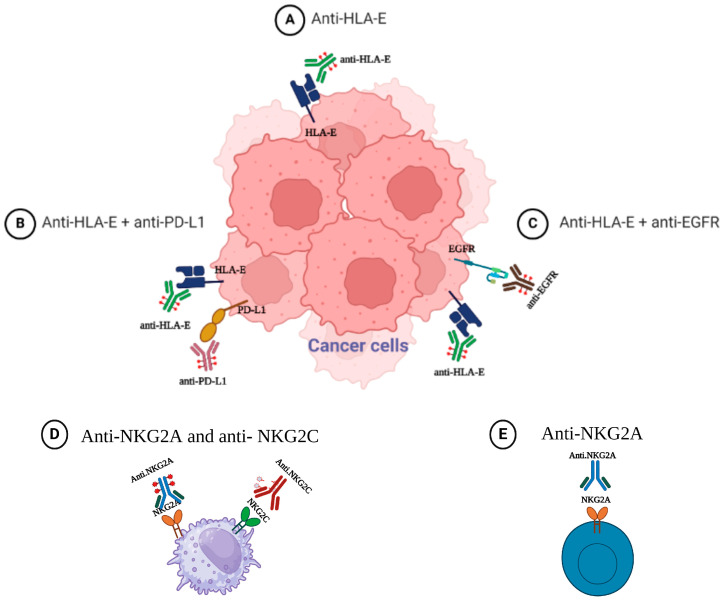
Potential therapeutic strategies to target HLA-E pathway.

In Figure 5 we illustrate potential therapeutic strategies for targeting the HLA-E pathway.

The main clinical trials targeting the HLA-E pathway are shown in Table 1.

**Table 1:** Clinical trials targeting the HLA-E pathway. CC, cervical cancer; CTLA4, cytotoxic T-lymphocyte associated protein 4; EGFR, epidermal growth factor receptor; ES-SCLC, extended stage small cell lung cancer; HLA, human leukocyte antigen; HER2, human epidermal growth factor receptor 2; mAb, monoclonal antibody; MPR major pathologic response; MSI/dMMR, microsatellite instability/deficient mismatch repair; MSS-CRC, microsatellite stable colorectal carcinoma; MSS-EC, microsatellite stable endometrial cancer; NKG2A, Natural Killer Group 2 member A; NA, not available NSCLC, non-small cell lung cancer; OC; ovarian cancer; ORR; overall response rate; PD-1, programmed cell death protein 1; PFS, progression free survival; HNSCC, head and neck squamous cell carcinoma. * Means estimated in the cited reference.

NCT	HLA-E Targeted Drug	In Combination with	Phase	Cancer Type	N (Accounted or Estimated *)	Status	Efficacy Results
NCT02671435 [102]	Monalizumab (anti-NKG2A mAb)	Durvalumab. (Durvalumab, cetuximab, mFOLFOX6, bevacizumab in a Part 3 MSS-CRC cohort)	1/2	MSS-CRC, NSCLC, MSS-EC, CC, OC	383 *	Active, not recruiting	Part 1 (dose escalation/expansion): ORR 0% Part 2 (dose expansion): ORR 10% (NSCLC), 7.7% (MSS-CRC); 5.4% (OC); 0% (MSS-EC)
NCT03088059 [101] * Cohort I1	Monalizumab	Alone (cohort I1), Durvalumab (cohort I2).	2	HNSCC	26 (Cohort I1); 340 * (total)	Active not recruiting. Terminated (cohort I1)	NA Cohort I1: ORR 0%
NCT02643550 [108]	Monalizumab	Cetuximab	1/2	HNSCC	143	Completed	No results posted
NCT04590963 [109]	Monalizumab	Cetuximab	3	HNSCC	264	Terminated	Terminated after interim analysis for futility
NCT02459301 [100]	Monalizumab	Alone	1	Gynecological	58	Completed	ORR 0%
NCT04307329 [110]	Monalizumab	Trastuzumab	2	HER2+ Breast cancer	11	Terminated	ORR 0%
NCT03822351 [103]	Monalizumab	Durvalumab	2, randomized	Stage III unresectable NSCLC	66 durvalumab arm; 61 combination arm.	Completed	ORR 35.5% vs. 17.9% 12 month PFS 72.7% vs. 33.9%
NCT03794544 [104]	Monalizumab	Durvalumab	2	NSCLC (neoadjuvant)	84 (total); 16 (durvalumab + monalizumab)	Completed	MPR rate 30% (combination) vs. 11.1% (durvalumab alone)
NCT03833440 [106]	Monalizumab	Durvalumab	2	NSCLC	120 (various arms) *	Active, not recruiting	NA
NCT05221840 [96]	Monalizumab	Durvalumab	3	Stage III unresectable NSCLC	999 *	Recruiting	NA
NCT03801902 [113]	Monalizumab	Durvalumab, radiation therapy	1	Locally advanced NSCLC	48 *	Recruiting	NA
NCT05061550 [105]	Monalizumab	Durvalumab, platinum doublet chemotherapy	2	NSCLC (neoadjuvant)	490 (various arms) *	Recruiting	NA
NCT05903092 [107]	Monalizumab	Durvalumab, platinum doublet chemotherapy	2	ES-SCLC	38 *	Recruiting	NA
NCT06152523 [114]	Monalizumab	MEDI5752 (Bispecific mAb, anti PD-1/CTLA4)	2	MSI/dMMR advanced cancers	43 *	Not yet recruiting	NA
NCT05162755 [115]	S095029 (anti-NKG2A mAb)	Sym021 (Anti-PD-1), anti-HER2 mAb, anti-EGFR mAb.	1	Solid tumors	51	Active, not recruiting	NA
NCT06094777 [116]	HY-0102 (anti-NKG2A mAb)	-	1	Solid tumors	50 *	Not yet recruiting	NA

### 6.2. HLA-G

HLA-G is scarcely expressed in healthy adult tissues, confined to immune privileged tissues such as the cornea, pancreatic islets, or erythroblasts [117,118,119]. Its immunosuppressive role has been widely studied in both healthy and neoplastic tissues, via its interaction with ILT-2 and ILT-4 (also known as human leukocyte immunoglobulin-like receptor B2 [LILRB1] and LILRB2, respectively), widely found on a variety of immune cells [120]. This, added to the fact that HLA-G is known to be over-expressed in most malignant tissues, makes it an immune checkpoint worth targeting for potential antitumor immune activation [15].

However, major limitations for HLA-G-targeted immunotherapy are its inter-patient, inter- and intra-tumor expression heterogeneity [121]. HLA-G is known to up-regulate PD-1 in tumor infiltrating T lymphocytes, leading to a potential antitumor synergy when combining anti-PD-1/PD-L1 antibodies with HLA-G targeted therapies [94]. Similarly, HLA-G seems to increase the expression of other immune checkpoints such as CTLA-4 and TIM-3 [94]. As discussed above, targeting the HLA-G pathway could hypothetically enhance the antitumor activity of ICIs and newer anticancer therapies. The use of antibodies, T cell engagers, CAR-T, and CAR NK cells for targeting the HLA-G pathway in cancer therapy could present both opportunities and challenges. Antibodies and T cell engagers targeting this pathway could potentially reduce tumor immune evasion [122]. However, the variability in HLA-G expression across different tumors could limit the efficacy of these therapies [9]. CAR-T and CAR-NK may offer a more robust response through their engineered specificity and persistence, yet the risk of off-target effects and cytokine release syndrome (CRS), in the case of CAR-T remains a significant concern [122,123].

A phase I clinical trial (NCT04485013) is testing the effects of the HLA-G inhibitor TTX-080 in various solid malignancies [124]. After a first dose escalation phase, a phase Ib dose expansion phase is being held testing the combination of the aforementioned TTX-080 with PD-1 inhibitor pembrolizumab or EGFR inhibitor cetuximab [124].

Anti ILT-2 antibody BND-22 has proven antitumor activity in the preclinical setting and is now under clinical development [125,126]. AGEN1571, another ILT-2 antagonist antibody, is being tested in a Phase I/II clinical trial (NCT05377528) both in monotherapy or in combination with a PD-1 inhibitor and/or a CTLA-4 inhibitor in patients with advanced resistant solid tumors [127].

The HLA-ILT4 (LILRB2) axis can be targeted with anti-LILRB2 mAbs. Results from the dose-escalation part of a phase I trial with the anti LILRB2 mAb MK-4830 showed a 24% overall response rate in the Pembrolizumab and MK-4830 combination arm in a variety of solid tumors, some of which had previously progressed to anti-PD-1/PD-L1 therapies [128]. MK-4830 and other anti-LILRB2 mAbs are also being tested alone or in combination with anti-PD-1 antibodies in a variety of solid tumors [128,129,130,131]. Another Phase I clinical trial (NCT05788484) is testing a PD-1 and LILRB2 bispecific antibody (CDX 585) [132].

JNJ-78306358, a CD3 HLA-G-targeted bi-specific T cell engager was evaluated in a Phase I clinical trial (NCT04991740) enrolling 39 patients with various cancers [133]. Common adverse effects were CRS and elevated liver enzymes, with dose-limiting toxicities in four patients leading to the halting of dose escalation due to these toxicities and the formation of anti-drug antibodies [133]. A Phase I/II trial (NCT05769959) in patients with HLA-G positive solid malignancies testing RO7515629, another T cell engager against HLA-G, was recently terminated by the sponsor [134].

HLA-G is also being evaluated as a promising antigen for chimeric antigen receptor (CAR)-T (or CAR-NK) therapy [19]. The efficacy and safety of anti-HLA-G CAR-T cells (IVS-3001) are currently being tested in a phase I/II trial with patients harboring HLA-G positive solid tumors (NCT05672459) [135]. However, one of the primary challenges in CAR-T development against solid tumors remains the significant toxicity CRS and the immunosuppressive TME [19]. These challenges could potentially be mitigated by precisely targeting immune checkpoint proteins, such as HLA-G, with CAR-NK therapy [19] or, with an even more innovative approach currently under clinical development, bispecific T cell engager-secreting HLA-G and PDL-1 targeted-CAR γδ T-cells (NCT06150885) [136].

**Figure 6:**
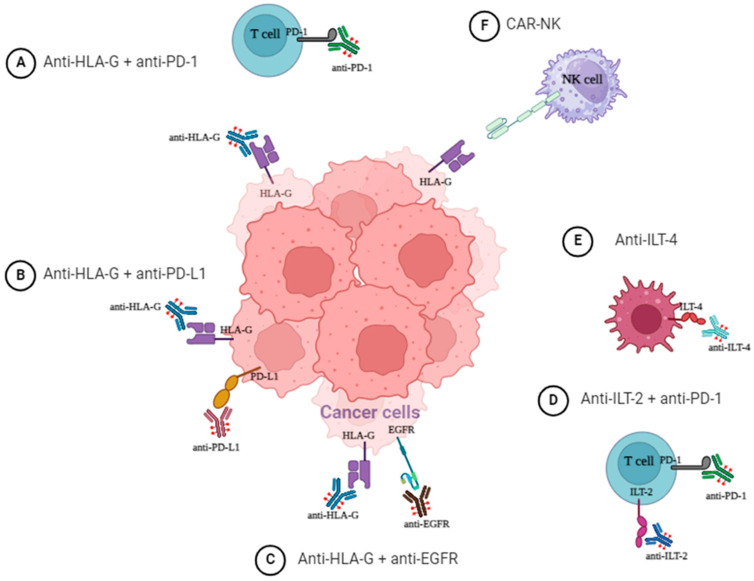
Potential therapeutic strategies to target HLA-G pathway.

In Figure 6 we illustrate potential therapeutic strategies to target the HLA-G pathway.

The main clinical trials targeting the HLA-G pathway are shown in Table 2.

**Table 2:** Clinical trials targeting the HLA-G pathway: BiTE, bispecific T-cell engager; CAR, chimeric antigen receptor; bsAb, bispecific antibody; CRC, colorectal carcinoma; ESCC, esophageal squamous cell carcinoma; ES-SCLC, extended stage small cell lung cancer; HLA, human leukocyte antigen; HER2, human epidermal growth factor receptor 2; ILT2, immunoglobulin-like transcription receptor type 2; LILRB2, leukocyte immunoglobulin like receptor B2; mAb, monoclonal antibody; MPR major pathologic response; MSI/dMMR, microsatellite instability/deficient mismatch repair; MSS-CRC, microsatellite stable colorectal carcinoma; MSS-EC, microsatellite stable endometrial cancer; NKG2A, Natural Killer Group 2 member A; NA, not available; NSCLC, non-small cell lung cancer; OC; ovarian cancer; ORR; overall response rate; PD-1, programmed cell death protein 1; PD-L1 (programmed death-ligand 1); PFS, progression free survival; RCC, renal cell carcinoma; HNSCC, head and neck squamous cell carcinoma; TNBC, triple-negative breast cancer. * Means estimated in the cited reference.

NCT	HLA-E Targeted Drug	In Combination with	Phase	Cancer Type	N (Accounted or Estimated *)	Status	Efficacy Results
NCT04485013 [124]	TTX-080 (anti-HLA-G mAb)	Alone, pembrolizumab, cetuximab	1	HNSCC, NSCLC, CRC, TNBC, RCC, melanoma	240 *	Active, not recruiting	NA
NCT04991740 [133]	JNJ-78306358 (HLA-G x CD3 BiTE)	Alone	1	CRC, OC, RCC	39	Completed	ORR 0%
NCT04717375 [126]	BND-22 (anti-ILT2 mAb)	Alone, pembrolizumab, cetuximab, carboplatin, pemetrexed	1/2	Solid tumors	456 *	Recruiting	NA
NCT05377528 [127]	AGEN1571 (anti-ILT2 mAb)	Alone, balstilimab, botensilimab	1	Solid tumors	22	Active, not recruiting	NA
NCT03564691 [128]	MK-4830 (anti-LILRB2 mAb)	Alone, pembrolizumab (dose escalation). Pembrolizumab, pemetrexed, Lenvatinib, paclitaxel, cisplatin (all arms)	1	Solid tumors	442 * Dose escalation: 84	Active, not recruiting	Dose escalation: ORR 24% (combination); 2% (monotherapy)
NCT05769959 [134]	RO7515629 (anti-HLA-G mAb)	Alone	1/2	HLA-G positive RCC, NSCLC, pancreatic adenocarcinoma, CRC, OC	3	Terminated	NA
NCT05672459 [135]	IVS-3001 (anti-HLA-G CAR-T cells)	Fludarabine phosphate, Cyclophosphamide	1/2	HLA-G positive solid tumors	117 *	Recruiting	NA
NCT05788484 [132]	CDX-585 (PD-1/LILRB2 bsAb)	Alone	1	Advanced solid tumors	130 *	Recruiting	NA
NCT04669899 [129]	JTX-8064 (anti-LILRB2 mAb)	Alone, pimivalimab (anti PD-1)	1/2	Solid tumors	190 *	Completed	Monotherapy: ORR 0%, SD 32%. Combination: ORR 11%; SD 33%.
NCT06007482 [130]	ES009 (anti-LILRB2 mAb)	Alone	1	Solid tumors	20 *	Recruiting	NA
NCT04165070 [137]	MK-4830	Pembrolizumab, carboplatin, pemetrexed, paclitaxel	2	NSCLC	360 *	Recruiting	NA
NCT04165083 [138]	MK-4830	Pembrolizumab	2	NSCLC (PD-L1+)	120 *	Active, not recruiting	NA
NCT04165096 [139]	MK-4830	Pembrolizumab	2	NSCLC	135 *	Active, not recruiting	NA
NCT04626518 [140]	MK-4830	Pembrolizumab	1/2	RCC	370 *	Active, not recruiting	NA
NCT04303169 [141]	MK-4830	Pembrolizumab	1/2	Melanoma (Neoadjuvant)	90 *	Active, not recruiting	NA
NCT04938817 [142]	MK-4830	Pembrolizumab	1/2	ES-SCLC (2L)	80 *	Active, not recruiting	NA
NCT04924101 [143]	MK-4830	Pembrolizumab, chemotherapy	2	ES-SCLC (1L)	120 *	Active, not recruiting	NA
NCT05319730 [144]	MK-4830	Paclitaxel, irinotecan, pembrolizumab, lenvatinib	1/2	ESCC	200 *	Recruiting	NA
NCT05342636 [131]	MK-4830	Pembrolizumab	1/2	ESCC	120 *	Active, not recruiting	NA
NCT05446870 [145]	MK-4830	Pembrolizumab, paclitaxel, carboplatin	2	OC (neoadjuvant)	160	Active, not recruiting	NA
NCT04895722 [146]	MK-4830	Pembrolizumab	2	MSI/dMMR-CRC	320 *	Recruiting	NA
NCT06380816 [147]	UCB4594 (anti-HLA-G mAb)	Alone	1/2	Advanced solid tumors	167 *	Not yet recruiting	NA
NCT06150885 [136]	HLA-G-CAR.BiTE allogeneic γδ T cells	Alone	1/2	Solid tumors	60 *	Not yet recruiting	NA
NCT06259552 [148]	SPX-303 (PD-1/LILRB2 bsAb)	Alone	1	HNSCC, RCC, CRC	232 *	Recruiting	NA
NCT06389526 [149]	CHS-1000 (anti-LILRB2 mAb)	Alone, toripalimab	1	Solid tumors	48 *	Not yet recluiting	NA
NCT06090266 [150]	OR502 (anti-LILRB2 mAb)	Alone, cemiplimab	1/2	Solid tumors	168 *	Recruiting	NA

### 6.3. HLA-F, HLA-H

The roles regarding tumor development and the progression of HLA-F and HLA-H are still unclear. Hence, it is not a surprise that these two proteins’ potential targetability has not been as explored as for their other two HLA Ib counterparts, HLA-E and HLA-G. New investigations could unveil a potential role for these two proteins in anticancer immunotherapy. For instance, preclinical observations suggest a potential role for anti-HLA-F antibodies in treating glioblastoma [18].

## 7. Future Perspectives and Conclusions

The study of non-classical HLAs in cancer has made significant strides in the last decade, offering new insights into tumor immunology and paving the way for innovative therapeutic approaches. Non-classical HLAs play significant roles in immunosuppression by interacting with inhibitory receptors on various immune cells [11,151]. HLA-E by interacting with CD94/NKG2A receptors on NK and T cells, inhibits their cytotoxic activity [13]. HLA-G binds to ILT2 and ILT4 receptors on T cells, NK cells, dendritic cells, and neutrophils, suppressing their immune functions, and promoting immune escape by tumor cells [15]. HLA-F, which binds to ILT2, ILT4, and KIR receptors on various immune cells, also contributes to immunosuppression by inhibiting immune cell activation and function [40]. HLA-H, while primarily known for its role in iron metabolism, can modulate immune responses by affecting the expression and function of other non-classical MHC class I molecules, such as HLA-E, thereby influencing NK and cytotoxic T cell activity [48]. These HLAs are predominantly present in immune cells, such as NK cells, T cells, and APCs [11]. Additionally, HLA-E is found in endothelial cells, HLA-F is present in B cells, monocytes, and trophoblasts, HLA-G is expressed in immune-privileged tissues such as the maternal-fetal interface of the placenta and pancreatic islet β cells, and HLA-H can impact other non-classical MHC class I molecules in various tissues [11,13,15,40,48].

Innate immunity constitutes a key aspect of the response against cancer in which non-classical HLAs play a pivotal role [95]. These molecules modulate the immune system by interacting with innate immune cells, particularly through NK cells, which are essential in the initial immune response against tumor cells [14,17,95,152]. NK cells can recognize and kill tumor cells that have downregulated classical HLA class I molecules, a common immune evasion strategy of tumors. However, non-classical HLAs can inhibit NK cell function [13]. By targeting these pathways, it is possible to restore NK cell function and enhance anti-tumor responses [16]. MDSCs represent another crucial component of the innate immune system that contributes to the immunosuppressive TME [79]. HLA-G has been shown to induce the expansion of MDSCs, which in turn suppresses T cell activity and promotes tumor growth [79]. By targeting HLA-G, we could potentially reduce MDSC-mediated immunosuppression, thereby enhancing the efficacy of other immunotherapies.

Therapies targeting non-classical HLAs offer promising but complex potential in cancer treatment. New therapeutic tools targeting non-classical HLAs are being tested, showing significant promise in this setting. These include bispecific antibodies, T cell engagers, CAR-T, and CAR-NK [19,94,132,135]. The benefits of these therapies include the ability to disrupt immunosuppressive interactions that these HLAs mediate, thereby restoring immune surveillance and enhancing anti-tumor responses [11]. For example, monoclonal antibodies targeting the HLA-E pathway, like monalizumab, have shown potential in enhancing the activity of NK and T cells by blocking inhibitory signals [16]. When combined with other immunotherapies like anti-PD-L1/anti-PD-1 antibodies, these therapies may achieve a more comprehensive immune activation [16]. However, there are limitations to these therapies. The efficacy of HLA-targeted treatments can be variable, and largely depend on the expression levels of HLAs in different tumor types and the heterogeneous nature of these tumors. This heterogeneity is highlighted by the activity of monalizumab principally in primary and not in metastatic tumors after chemotherapy, or, on the other hand, the activity of anti-LRLB2 antibodies in tumors that have progressed to anti-PD1 therapies. Additionally, targeting HLAs can lead to immune-related adverse effects, including the risk of off-target effects and CRS, particularly with CAR-T [122,123].

Research in non-classical HLAs in cancer over the past decade has set a foundation for future studies and clinical applications. These molecules’ diverse roles in immune modulation and tumor progression open new avenues for personalized cancer therapies and diagnostics. As our understanding of these complex molecules continues to grow, so does the potential for developing more effective and targeted cancer treatments, making this an exciting and promising field of study in oncology.
